# Present and future thermal environments available to Sharp-tailed Grouse in an intact grassland

**DOI:** 10.1371/journal.pone.0191233

**Published:** 2018-02-07

**Authors:** Edward J. Raynor, Larkin A. Powell, Walter H. Schacht

**Affiliations:** 1 School of Natural Resources, University of Nebraska-Lincoln, Lincoln, NE, United States of America; 2 Department of Agronomy and Horticulture, University of Nebraska-Lincoln, Lincoln, NE, United States of America; Sichuan University, CHINA

## Abstract

Better understanding animal ecology in terms of thermal habitat use has become a focus of ecological studies, in large part due to the predicted temperature increases associated with global climate change. To further our knowledge on how ground-nesting endotherms respond to thermal landscapes, we examined the thermal ecology of Sharp-tailed Grouse (*Tympanuchus phasianellus*) during the nesting period. We measured site-specific iButton temperatures (T_iB_) and vegetation characteristics at nest sites, nearby random sites, and landscape sites to assess thermal patterns at scales relevant to nesting birds. We asked if microhabitat vegetation characteristics at nest sites matched the characteristics that directed macrohabitat nest-site selection. Grouse selected sites sheltered by dense vegetation for nesting that moderated T_iB_ on average up to 2.7°C more than available landscape sites. Successful nests were positioned in a way that reduced exposure to thermal extremes by as much as 4°C relative to failed nests with an overall mean daytime difference (±SE) of 0.4 ±0.03°C. We found that macrohabitat nest-site selection was guided by dense vegetation cover and minimal bare ground as also seen at the microhabitat scale. Global climate projections for 2080 suggest that T_iB_ at nest sites may approach temperatures currently avoided on the landscape, emphasizing a need for future conservation plans that acknowledge fine-scale thermal space in climate change scenarios. These data show that features of grassland landscapes can buffer organisms from unfavorable microclimatic conditions and highlight how thermal heterogeneity at the individual-level can drive decisions guiding nest site selection.

## Introduction

Animals inhabit thermal environments that place constraints on physiological processes [1] and mediate the success of important life history stages [2–4]. Accordingly, animals employ behavioral and physiological mechanisms to counteract thermal restrictions on specific life history stages [5–7]. For example, precocial, ground-nesting birds in grasslands (e.g., grouse), regularly face unfavorable environmental conditions, such as extreme heat, during incubation that may influence egg and nest survival. As determinant breeders, they do not incubate their eggs until their clutch is complete; thus eggs are exposed to environmental stressors before parents initiate incubation [4]. Unfavorable environmental conditions may cause stress on incubating adults, resulting in additional time away from the nest to meet their own physiological demands (e.g., acquiring water or food), leading to decreased nest attentiveness or possibly abandonment [8, 9]. Nest survival may be affected by the incubating adult’s ability to choose nest sites that minimize predation risk, meet their physiological needs, and protect eggs from the elements [9].

Animals adapt to their thermal environment in various ways depending on their body size, mobility, and stage of life [2, 10, 11]. Behavioral decisions to meet thermal-based physiological demands are usually made at fine temporal scales [3, 12–14]. However, studies of thermal conditions often focus on spatial and temporal scales at coarser resolution [15, 16]. Such discrepancies can hinder our understanding of individual-level reactions to subtle, fine-scale changes in thermal conditions, reducing our ability to assess species’ responses to environmental change [17–19].

Vegetation structural heterogeneity is frequently a driver of fine-scale behavioral decisions by animals, such as patch selection [20]. Ecological investigations of the effects of habitat heterogeneity have usually focused on spatiotemporal variability in vegetation structure, without addressing its associations, such as the underlying microclimate [21]. Spatial and temporal variation in near ground thermal conditions creates thermal heterogeneity within landscapes and directs behaviorial and physiological processes, such as thermoregulation [22, 23]. Thermal refugia within heterogeneous landscapes are microhabitats that buffer extreme thermal conditions (e.g., oppressive temperature and humidity) for nesting activities [7, 21, 24]. Therefore, how these local thermal environments modulate animals’ behavioral decisions is a matter of biological significance [15].

Our lack of understanding of thermal environments may slow conservation efforts in cases where species’ persistence depends on appropriate thermal space [24–26], particularly when considering projections for global climate change [27]. This knowledge gap can be filled by evaluating thermal habitat selection in a thermal context at the scales animals experience during critical life stages [2, 10]. Assessment of abiotic and biotic factors may provide insight into the underlying basis for thermal habitat selection [28, 29].

For birds, the selection of a nest site is a decision that in part determines the eggs’ thermal environment with critical impact on chick development and survival [22, 30, 31]. Nest structural characteristics and adult incubation activities are essential for nest success [24, 26, 32–34], and nest microsite characteristics are critical for providing physiological relief from abiotic thermal factors that can limit reproduction [3, 21]. The choice of a nest site is also tied to minimizing predation risk [35]. Thus, birds will choose nest sites that minimize predation risk and exposure to unfavorable thermal conditions.

Ground-nesting gallinaceous birds, such as Northern Bobwhite (*Colinus virginianus*), are vulnerable to thermal extremes [2, 3, 36, 37]. For example, Guthery, Rybak [38] reported that signs of heat stress in bobwhite included gular flutter near 31°C air temperature. Gular flutter is a physiological mechanism used to prevent and regulate hyperthermia via evaporative cooling of water on the surface of the respiratory tract, which is commonly used by Galliforms during periods of high heat exposure [39, 40]. This behavior is costly to females because of the associated water loss [41, 42]. Galliforms obtain water largely through metabolic water and pre-formed water in food. When experiencing 46°C air temperature for at least 1 hr bobwhite eggs incur 50% mortality [43]. Adults and chicks face reduced performance and population declines as the result of periods of extreme heat [38]. Solar radiation and air temperature have been shown to be important predictors of nest survival for two grouse species, Greater Prairie-Chicken (*Tympanuchus cupido*; [21]) and Lesser Prairie-Chicken (*Tympanuchus pallidicinctus*; [24, 26]) in the southern Great Plains of North America. To our knowledge, no studies have assessed the thermal aspects of grouse reproductive ecology in the northern Great Plains. Investigations of the thermal ecology of the regionally common Sharp-tailed Grouse (*Tympanuchus phasianellus*), are notably lacking [44], which is a cause for concern as this species’ range is contracting [16, 44]. Sohl (16) predicted at least a 25% reduction in Sharp-tailed Grouse range by the year 2075 in an analysis that evaluated broad-scale climate and land cover change.

**Fig 1:**
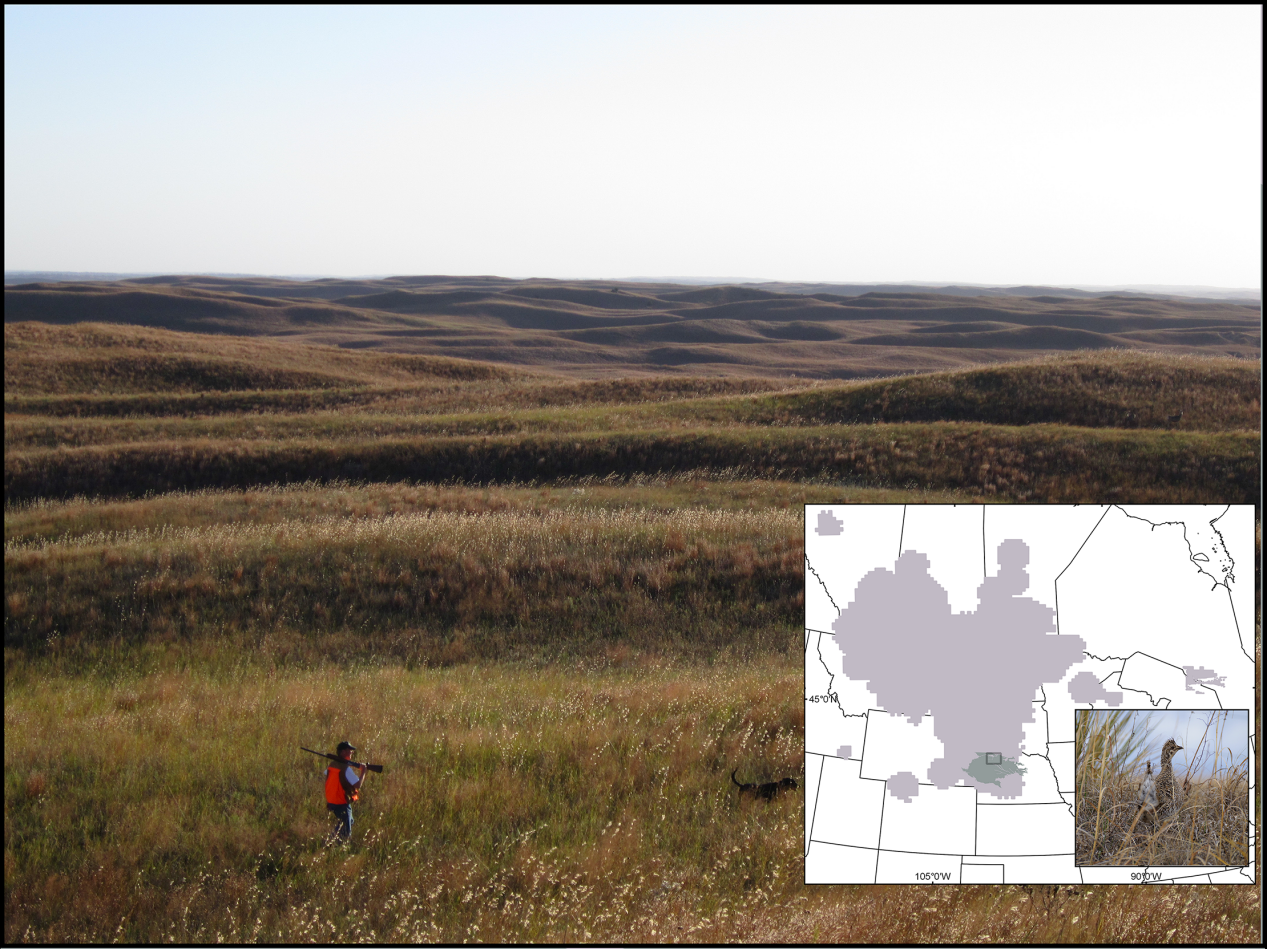
Representative topography of the Nebraska Sandhills in north-central Nebraska, U.S.A; photograph by Eric Schacht. Inset: Sharp-tailed Grouse distribution in the central United States and central Canada (gray) with Nebraska Sandhills in dark gray and study area in box, photograph of Sharp-tailed Grouse taken by Joel Jorgenson.

Because organisms face risks associated with thermal stress when selecting habitat among heterogeneous landscapes [1, 22, 45], a critical need exists to understand the thermal heterogeneity of landscapes. We examine thermal heterogeneity of microhabitats available to a grassland nesting bird, Sharp-tailed Grouse (hereafter grouse, Fig 1, inset). We hypothesize that in a setting where air temperatures can reach levels to cause thermal stress, 1) grouse would select nest locations offering structural characteristics that would moderate microclimatic conditions and 2) future climate change predictions would constrict the operational thermal space for grouse. We examined thermal and vegetation structural characteristics of nest sites relative to random selected sites within 3 m of the nest, and available landscape sites to evaluate the role of thermal conditions on nest habitat choices and to identify the extent of variation in thermal properties. To understand how vegetation characteristics critical to microhabitat selection mediated nest site choices at a coarser within-pasture scale (within 150 m of the nest), we evaluated the role of vegetation characteristics in guiding macrohabitat nest-site selection decisions. Finally, to gain deeper insight into the potential role of climate change in impacting nest microclimate, we considered the extent to which thermal conditions at contemporary nest locations may change when exposed to increased temperatures. Such fine-scale information can advance the understanding of underlying mechanisms responsible for predicted distributional changes.

## Materials and methods

### Study area

We examined grouse thermal ecology in the expansive mixed-grass prairie ecosystem of the Nebraska Sandhills, an area spanning over 4,998,000 ha [46] in north-central Nebraska, U.S.A. This grassland is located on the south-eastern periphery of the grouse distribution in the Great Plains (Fig 1 inset; [16, 47]). The study site encompasses 75,916 ha and was located on the Valentine National Wildlife Refuge (28,941 ha; 42.5129° N, 100.5521° W) and Samuel R. McKelvie National Forest (46,975 ha; 42.7190° N, 101.0248° W) which are managed by the U.S. Fish and Wildlife Service and the U.S. Forest Service, respectively. Plant communities in Nebraska Sandhills grasslands include the bunchgrass community and is dominated by the warm-season tallgrasses including *Schizachyrium scoparium*, *Calamovilfa longifolia*, *Panicum virgatum* and *Andropogon halii* [46]. Although grasses make up the dominant cover type in the Sandhills, there are numerous species of forbs and some shrub species [48]. The Sandhills are semi-arid and subject to variation in annual precipitation, receiving approximately 50cm of precipitation annually [46]. The topography is characterized by mostly linear dunes averaging between 41–50 m in height (mean elevation: 970m asl), with south- and southwest-facing slopes having steep inclines, ranging from 20–28 degrees (Fig 1; [49]). Because of the extensive sandy soils there is little row crop cultivation on or near the study site. Agricultural land use in this region consists of haying and low-intensity cattle grazing (e.g., Valentine NWR stocking rate mean is 0.28 Animal Unit Months (AUM) ha^-1^ with a range of 0.24 to 1.3 AUM ha^-1^, Melvin Nenneman, personal communication). Other public grazing lands in this region have stocking rate means of 0.52 AUM ha^-1^, whereas mean stocking rate may be higher on private lands (e.g., 0.98 AUM ha-1; [50]).

### Data collection

Adult female grouse were captured using walk-in traps [51] on leks from March to May 2015–2016 and fitted with a 16g VHF radio-transmitter (Advanced Telemetry System, Isanti, Minnesota, USA). We released each bird immediately after processing at the capture location. We monitored 23 nests located by tracking female grouse during the nesting season to determine nest outcome (i.e., successful or failed). In 2015, 16 nests were located; we refer to these nests only in our macrohabitat nest-site selection analyses because 2015 nest temperatures were not recorded. We minimized the amount of time spent at the nest (< 5 min) and revisited a nest to assess nest fate only when we verified that the female was off the nest. At each nest site, we counted the number of eggs present at first discovery and monitored nests every 3–5 days until fate was established. We considered nests successful if ≥ 1 egg hatched.

We measured air temperature to quantify the thermal environment of nests and the local landscape. To quantify thermal environments at the landscape scale, we recorded air temperature using a Maxim Integrated data logger (Maxim Integrated Products, Sunnyville, California, USA; hereafter “iButton”) placed at ground-level. Manufacturing specifications include: temperature accuracy of ± 0.5°C from -10 to +65°C, and operating range -40°C to +85°C. We used iButton measurements with caution as they differ from grouse and their eggs in size, volume, and surface area, which may affect the device’s ability to scale temperature, solar radiation, and convection values to body size [52, 53]. Sampling periods were one week long and conducted twice during the nesting season (i.e. mid-May and mid-July). To capture spatial variation in air temperature, we used five 50 m transects that ran horizontally along the southern base, southern slope, top, northern slope, and northern base of dunes. Within each transect, iButtons were randomly placed within a 2m circle centered at 0 m, 25 m, and 50 m along the transect; thus, there were 3 sampling points per transect, totaling 15 sampling points per dune. We replicated the landscape grid on four dunes of varying elevation for both sampling periods to describe spatial variation and develop our thermal landscape characterization. Dunes were selected within our study area using a stratified random sampling approach in ArcGIS 10.3 (Environmental Systems Research Institute, Redlands, California, USA).

We measured air temperature at each nest site on the predicted hatch date (i.e., start of incubation plus 23 days [44] by placing one iButton in the nest bowl and three iButtons at randomly selected locations within 3m of the nest bowl. iButton air temperatures (hereafter, T_iB_) were recorded every ten minutes with a resolution of 0.0625°C for a 24-hour period at 23 nests. The forecasted hatch date was used to standardize deployment times at failed (n = 14) and successful (n = 9) nests [3, 21]. Grouse nests are composed of dead grasses and forbs, dead leaves, and lined with vegetation and feathers; however, the role of these materials in mediating nest microclimate is unknown [44]. Because Sharp-tailed Grouse select nest sites in patches of grassland dominated by dead plant material [54], we assume the thermal environment of the nest site during the post-hatching period of our thermal measurements is not affect by progression of leaf out.

We recorded the vegetation parameters shrub, grass, forb, bare ground, and litter coverage in 10% classes on a 0.5 m^2^ quadrat centered over the iButtons both at the nest bowl and at the 3 associated random sites. We measured vegetation height (dead and live) and litter depth at each iButton and took visual obstruction readings (VOR) to the nearest 0.25 dm from a distance of 4 m and a height of 1 m, using a Robel pole placed at the nest bowl [55]. To understand vegetation characteristics important for nest-site selection within the same pasture at the macrohabitat scale [56], we randomly selected 5 points within 150m of the nest for comparison with nest vegetation characteristics. At this macrohabitat scale, we measured the same vegetation characteristics that were measured in the immediate area of the nest but also VOR, which permitted us to assess the role of vegetation structure in driving within pasture, nest-site selection.

### Statistical analysis

To compare site-specific T_iB_ measurements with concurrent macroclimate variables, we modeled hourly T_iB_ at grouse nest locations and across the landscape based on ambient air temperature and vapor pressure deficit (VPD) [24]. The macroclimate variables, ambient temperature (T_air_) and relative humidity were recorded hourly at 2 m above ground-level at Miller Airfield, Valentine, Nebraska (42°51′24″N 100°32′56″W); this location is < 12 km from the study area. To better illustrate ambient conditions, we calculated the VPD, the difference between the amount of moisture in the air and how much moisture the air can hold when saturated (mmHg), by using the paired hourly ambient temperature and relative humidity measurements from the weather station [57]. VPD is considered a better measure of aridity than relative humidity [57]. Because site-specific T_iB_ were not all recorded on the same dates, we used our models to predict air temperatures at grouse nests and across the landscape on the days that air temperatures were measured at nests, random microsites, and landscape sites. This modeled data were used when comparing air temperatures and trends among nests, nearby random microsites, and across the landscape.

As thermal stress has not been previously evaluated for grouse in the northern Great Plains, we used thermal thresholds developed for bobwhite and Lesser Prairie-Chicken from field studies in the southern Great Plains [24, 38]. Lesser Prairie-Chicken daily nest survival probability begins to decrease by 10% every half-hour at air temperatures greater than 34°C [24] and bobwhite begin to exhibit thermal stress at 31°C [38]. Furthermore, to assess whether microclimates of failed or successful nests exceeded 46°C for more than an hour as a measure of thermal stress, we calculated the amount of time each nest experienced this level known to induce mortality in Galliform eggs [43].

We examined variation in vegetation measurements among nest and nearby random sites with separate linear mixed-effects (LME) models for each vegetation parameter using the *lmer* function in the lme4 package [58] in R version 3.2.3. We assessed whether vegetation composition differed between nests and random sites by comparing dead standing vegetation height, live grass standing height, litter depth, and percent cover of bare ground, grass, litter, forbs, and shrubs between site types. Individual nest identifier (categorical) was included in the models as a random effect to account for multiple observations of nests from a single individual. Two nests for one female that re-nested were measured and included in the analysis. We examined variation in VOR at nests that hatched and failed; VOR was not measured at nearby random microsites.

We analyzed within pasture, macrohabitat selection of nesting grouse by comparing the vegetation measurements of each nest with the same features taken at 5 random points within 150 m of the nest and in the same field. At this scale, we used discrete choice models to assess the probability of female grouse selecting nest sites based on characteristics of the used and available resources [59]. Prior to our discrete choice analysis, we were prepared to remove predictor variables if their correlation coefficients exceeded 0.4. Bare ground cover and VOR (*r* = -0.44) were the only variables exhibiting multicollinearity; these variables were not used together in subsequent analyses. We compared nesting sites to paired random sites using conditional (i.e., case-controlled) logistic regressions, using *clogit* in the R survival package with the paired used and available site identifier as the strata term [60, 61]. We created four biologically reasonable *a priori* models to explain the response variable, whether the site was used for nesting (coded as 1) or not (coded as 0). These models included a null model, using the following sets of covariates: a quadratic model of vegetation composition (% cover; shrub + shrub^2^ + standing dead vegetation + standing dead vegetation^2^ + bare ground + bare ground^2^), a quadratic model of vegetation structure (VOR + VOR^2^ + litter depth (cm) + litter depth^2^ + max. vegetation height (cm) + max. vegetation height^2^), and a global model (cover and structure). We expected vegetation composition could be a key component in macrohabitat selection [62, 63], but vegetation structure may become too dense for nesting habitat (as measured by VOR, litter depth and maximum vegetation height; [64]). We used quadratic polynomial terms for vegetation structure variables because of the possiblility of avoidance of extreme vegetation densities as suggested by Buhnerkempe, Edwards [65]. Models were ranked on Akaike’s information criterion corrected for small sample size (AIC_*c*_). For each model *i*, we calculated the difference between the AIC_*c*_ of model *i* and the AIC_*c*_ of the best model (ΔAIC_*c*_) and Akaike weight (ω_*i*_). Models with ΔAIC_*c*_ < 2 were considered to have substantial empirical support [66]. When we hypothesized a non-linear relationship, we performed a preliminary comparison of a linear model and a non-linear model before continuing with the global analyses. For all model comparisons, we created a confidence set of models with a combined model weight of ≥ 90% [66]. We were prepared to choose the top model if it was the most parsimonious; otherwise we were prepared to model-average. We plotted effects from the top model using 85% confidence intervals to match the inference provided by model comparisons with AIC_*c*_ [67]. This macrohabitat analyses used vegetation data from 39 grouse nests monitored in 2015 (n = 16) and 2016 (n = 23).

To examine potential future changes in thermal space, we used linear regression of T_iB_ and T_air_ measurements to project T_iB_ under future climate scenarios. To characterize changes in nest site and landscape microclimates related to predicted climate change, we used simple linear model outputs instead of adding predicted increases onto observed T_iB_ [3]. Projected T_air_ used to model future T_iB_ were obtained by averaging global climate models for low and high carbon dioxide emission scenarios predicted for our study area at the end of century (2080) (www.climatewizard.org; [3, 27, 36]). Average T_air_ at our study area are predicited to increase by 2.8°C and 4.5°C for low and high end of the century emission scenarios, respectively. We compared our predictions of T_iB_ at nests and landscape sites to determine whether 2080 temperatures differed across the same ranges of observed T_iB_ [3], thus providing comparison of relative thermal conditions between observed and future conditions. Unless otherwise noted, means are reported ± 1 SE and significance is set at α = 0.05. All statistical analyses were performed in Program R 3.2.3 [60].

### Ethics statement

Permission to capture and monitor grouse was granted by the United States Fish and Wildlife Service and the United States Forest Service, which manage the Valentine National Wildlife Refuge and Samuel R. McKelvie National Forest, respectively. The University of Nebraska-Lincoln Institutional Animal Care and Use Committee approved our grouse capture and handling procedures (IACUC #901 and #1265).

## Results

**Fig 2:**
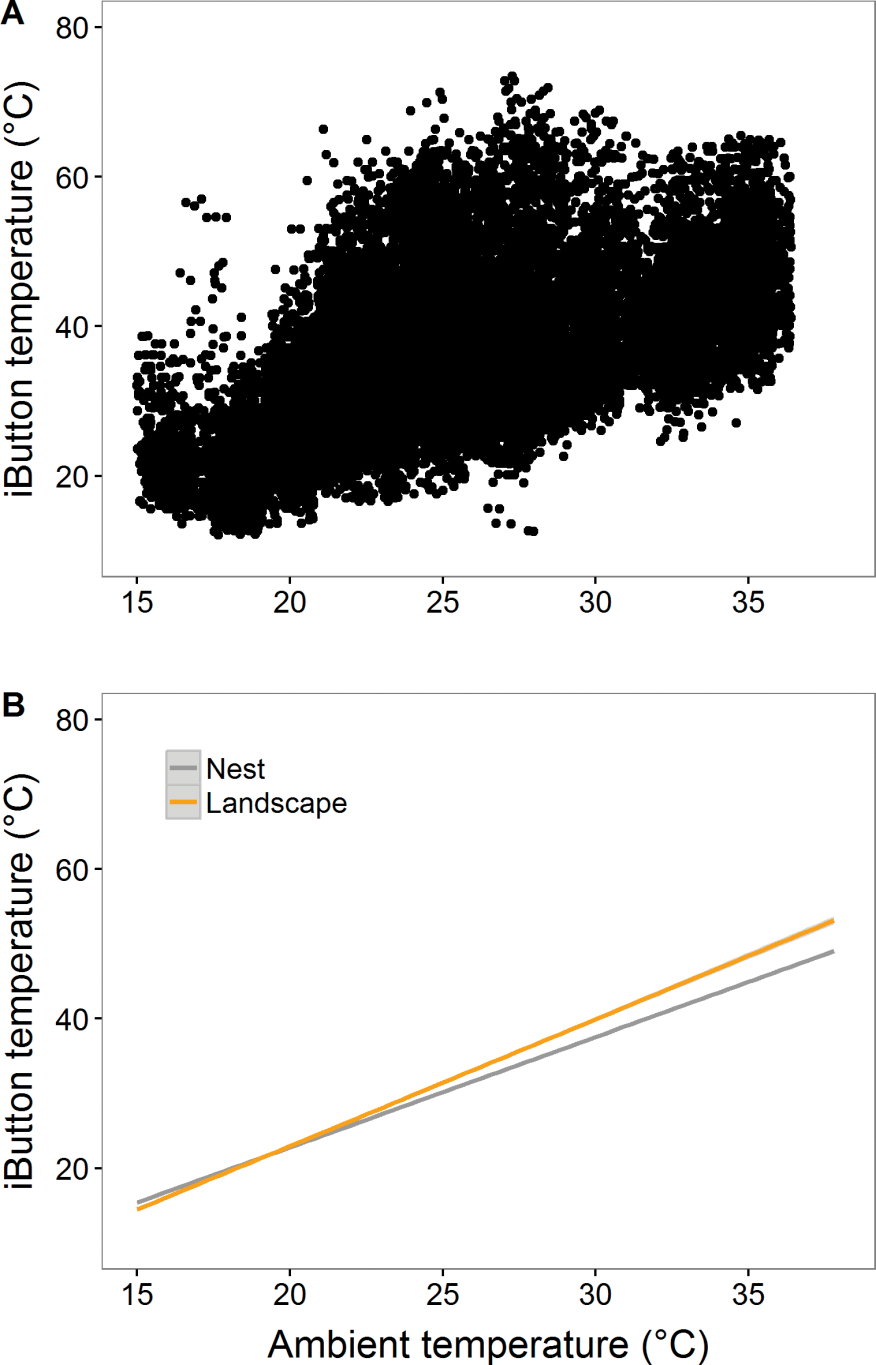
Grouse nests moderate thermal environments; an observation that is particularly evident during periods of extreme heat. (A) Temporal spectrum of all iButton temperature measurements recorded during 09:00–19:00 h and (B) results of simple linear models for iButton temperatures (T_iB_) explained by air temperature (T_air_) measurements collected during the full 24 h period at nest sites and landscape points for Sharp-tailed Grouse at the Nebraska Sandhills, Valentine, Nebraska, USA (May to July, 2016).

Within this grassland landscape, nest sites moderated T_iB_ markedly, with surrounding locations on the landscape (mean ± SE T_iB_: 21.10±0.21) exhibiting T_iB_ > 60°C in 25 locations during early July, whereas only eight of the 23 nest sites experienced this extreme temperature. We determined that the landscape exhibited microclimatic heterogeneity with T_iB_ differences ranging in time and space by as much as 40°C, which was evident when T_air_ was greater than 28°C (Fig 2A and S1 Data). Multiple generalized linear regression (GLM) models of T_iB_ for nest and random sites revealed T_air_ and VPD recorded at a meteorological station and their statistical interaction were useful in explaining variation in T_iB_ measurements (~ 70%) (Table 1).

**Table 1:** Fit and parameter estimates (± SE) from multiple regression (GLM) models for relationships of thermal environments at nest sites, random sites (within 3 m), and nests that hatched or failed with air temperature (T_iB_) and vapor pressure deficit (VPD) in the Nebraska Sandhills, Valentine, Nebraska, USA (2016).

Site Modeled	Intercept	T_air_	VPD	T_air_ X VPD	Fit (R^2^)
Nest*	4.20 (± 0.79)	0.70 (± 0.05)	0.70 (± 0.08)	-0.01 (± 0.002)	0.69
Random*	3.80 (± 0.26)	0.50 (± 0.02)	1.61 (± 0.03)	-0.03 (0.001)	0.73
Hatch nest*	4.63 (± 1.25)	0.75 (± 0.07)	0.35 (± 0.04)		0.69
Fail nest*	4.79 (± 0.97)	0.68 (± 0.06)	0.51 (± 0.04)		0.70

* Indicates significance at a level of P < 0.01.

### Microsite characteristics

**Fig 3:**
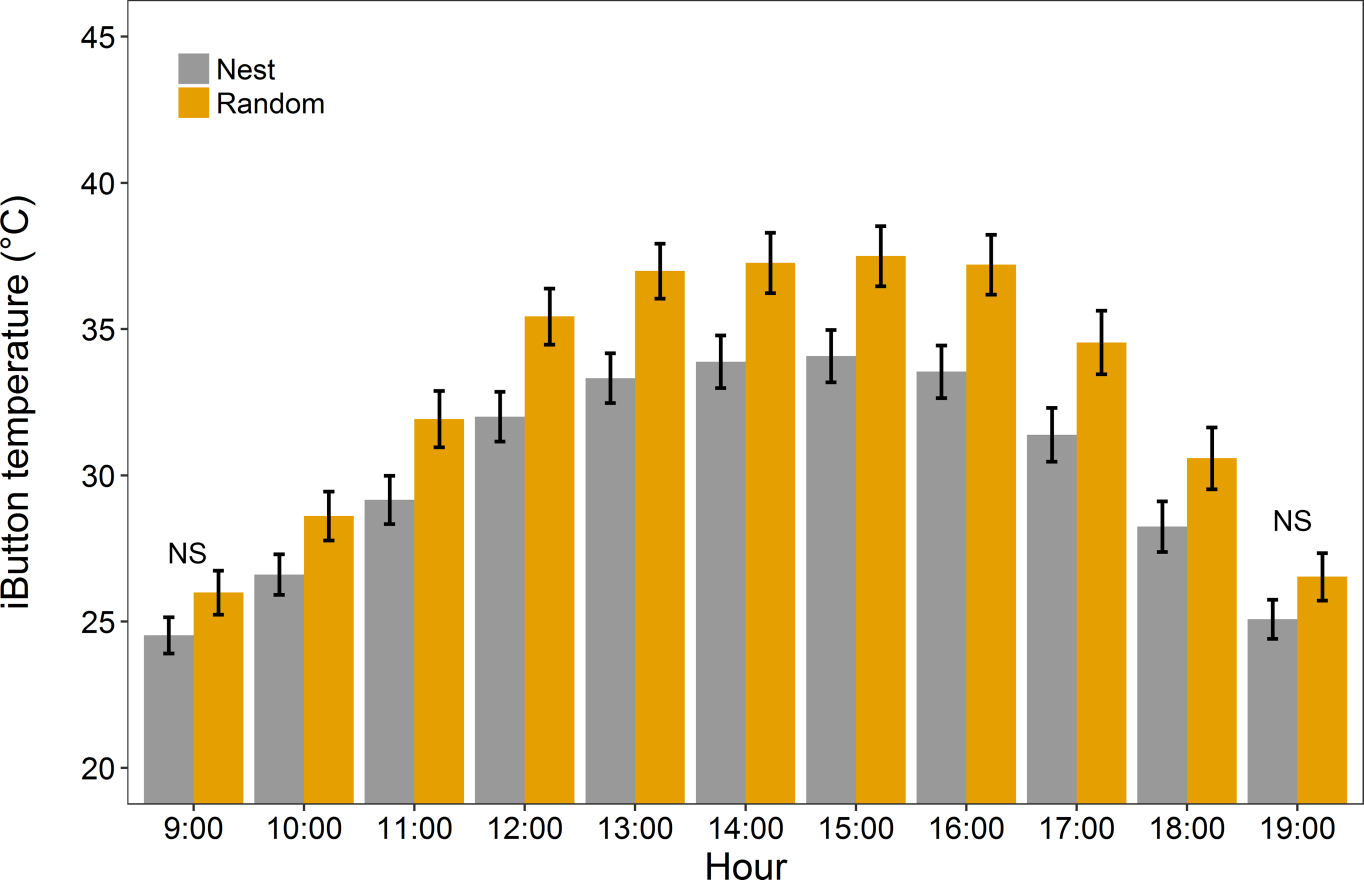
Nest sites are buffered from thermal environments within 3 m at random microsites. Mean iButton temperatures (T_iB_) (± SE) recorded at Sharp-tailed Grouse nest sites (gray) (n = 23) and random sites (orange) (n = 69) from the daytime period (09:00–19:00 h) in June 2016.

Mean 24-hr temperatures (±SE) for nest and random sites were 23.66 (0.18) and 24.76 (0.22). Nest sites buffered their contents against air temperature observed on the landscape by exhibiting slightly warmer T_iB_ when T_air_ was < 20°C (Fig 2B); yet cooler T_iB_ than landscapes at T_air_ > 20°C. The difference of T_iB_ and T_air_ (e.g., T_iB_−T_air_) at nest sites was reduced by about a third as much as landscape sites (2.7°C) during the day (09:00–19:00 h; S1 Fig). While nest locations moderated nest thermal conditions to a greater extent than the available habitat on the landscape, extreme heat potential (i.e., oppressive T_iB_) at nests was also evident. Unoccupied nests were exposed to daytime temperatures above 31°C for 7.33 (0.28) hours from 09:00–19:00, whereas nearby microsites reached at least 31°C for 8.77 (0.19) hours during the same time period; suggesting that nests were placed in locations that are less likely to expose birds to conditions requiring evaporative cooling. Our expectation that nest sites would offer time-specific thermal buffering was met; we found temporal differences in T_iB_ at nest and nearby random sites within 3 m across the day (09:00–19:00) with differences being substantial during 11:00–16:00 h (Fig 3). Differences in T_iB_ at nest sites and random sites reached their maximum during the afternoon period. With the exception of 09:00 and 19:00, nests exhibited lower temperatures than nearby microsites throughout the day (ANOVA time x location interaction, P <0.0001).

**Fig 4:**
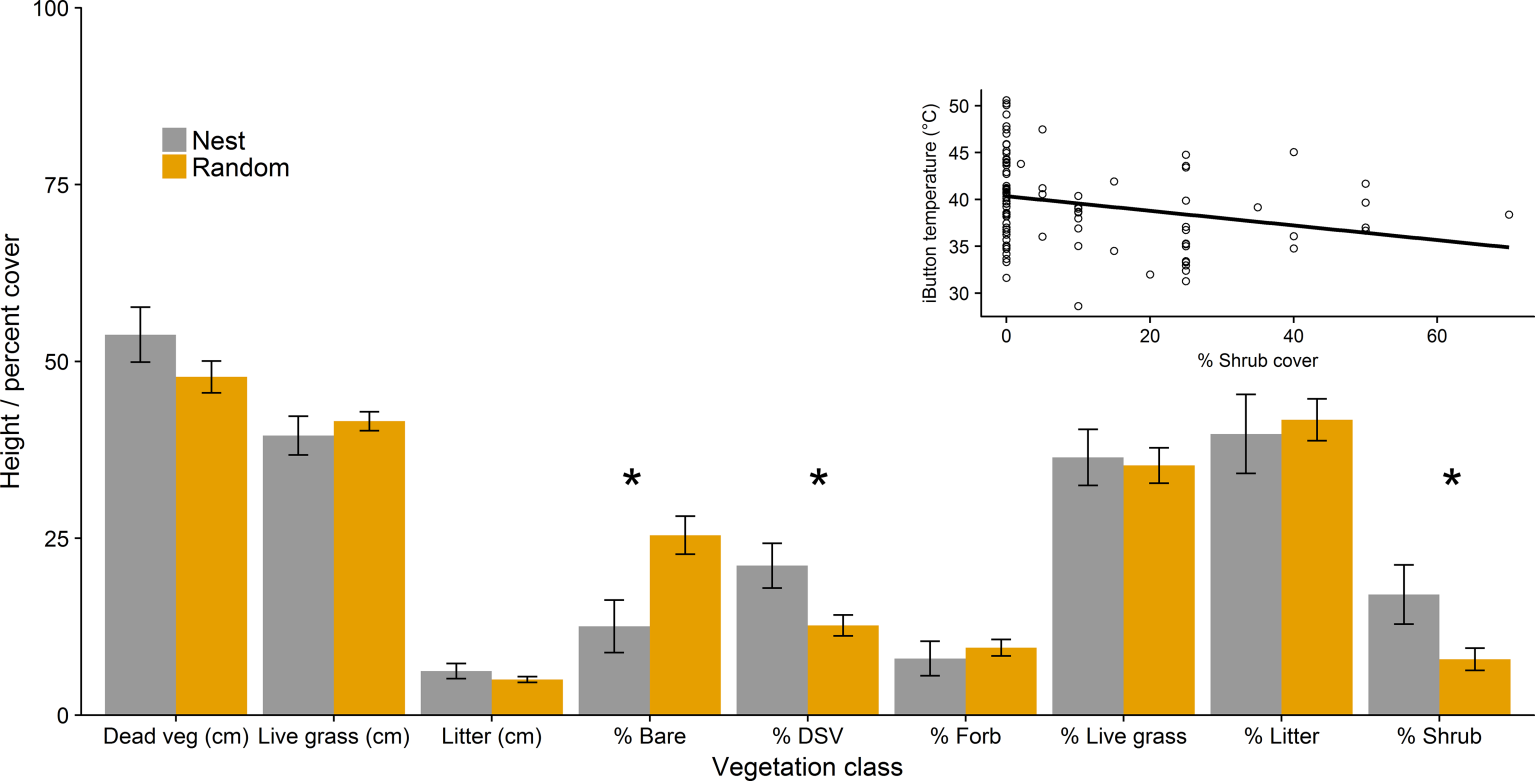
Vegetation characteristics at the microsite scale vary among nest and nearby random microsites. Vegetation characteristics (±SE) measured at nest and pooled random sites in the central Nebraska Sandhills, Valentine, Nebraska, USA in 2016. Inset is diurnal T_iB_ plotted over increasing shrub cover at nest and random sites. An asterisk denotes significance at α = 0.05.

**Fig 5:**
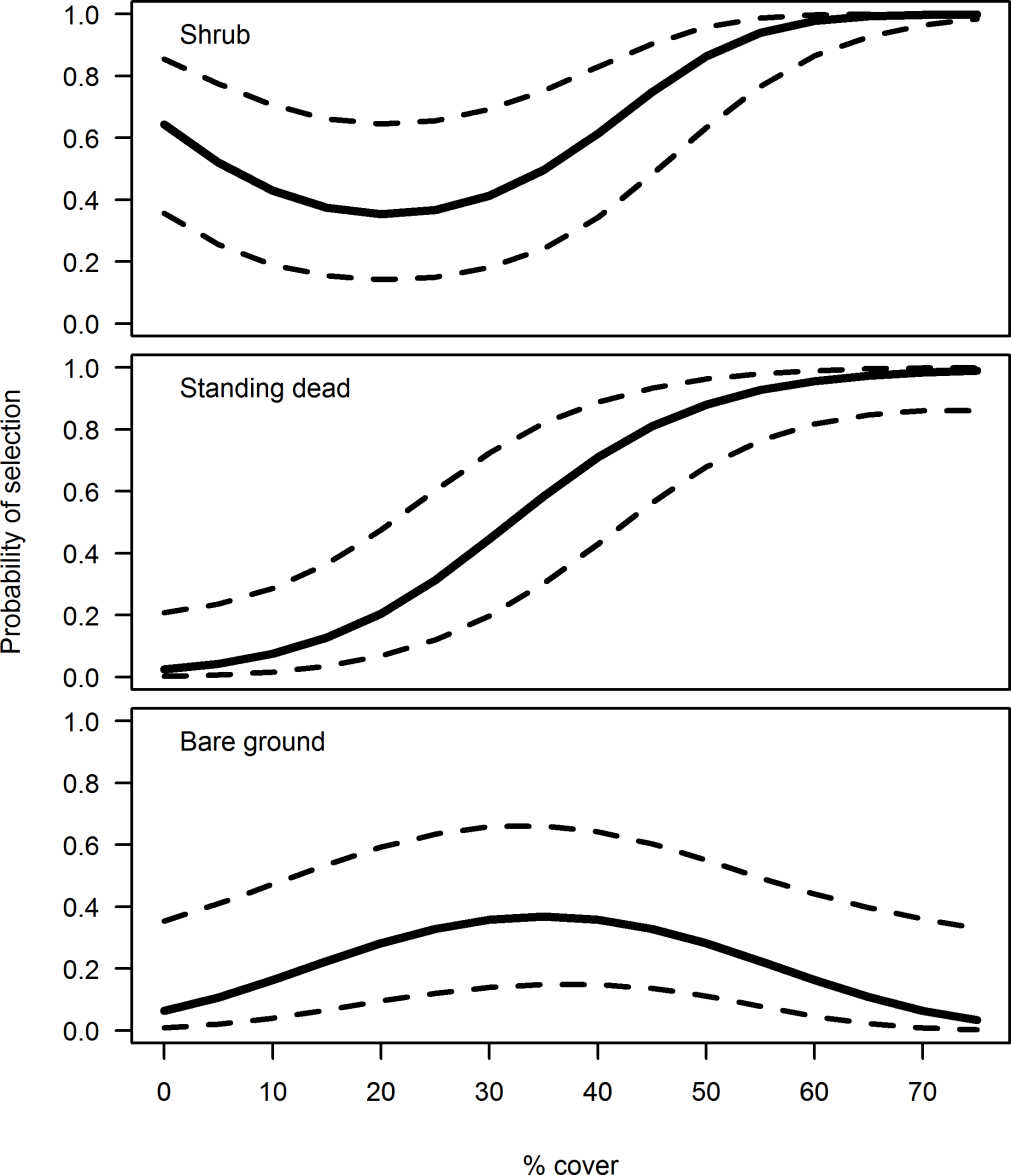
Within pasture nest site selection demonstrated an affinity for dense cover. Probability of selection with 85% confidence intervals, as a function of covariates in the best macrohabitat discrete choice model, vegetation composition, by nesting sharp-tailed grouse in the central Sandhills of Nebraska, 2015–2016. Predictions of selection for shrub cover, standing dead vegetation cover, and bare ground cover are shown for the range at those measurements at our study site. All variables not plotted were held constant at their means to show variation in the covariate of interest.

Nest site daytime T_iB_ (mean ± SE: 26.99 ± 0.11, range: 8.08–68.44, n = 23) had a narrower range and lower mean than random sites (28.65 ± 0.24, range: 6.60–76.23, n = 69). This difference between random sites and nests at such fine scales shows the apparent thermal variability within mixed-grass prairie. Vegetation measurements at nest and random sites were similar for all parameters with the exception of bare ground cover being lower at nests (F_1,68_ = 10.79, P = 0.002; Fig 4), while shrub cover (F_1,68_ = 8.70, P = 0.004) and standing dead vegetation cover (LME; F_1,68_ = 10.94, P = 0.002) was greater at nests; shading by shrubs and standing dead could be driving nest temperatures. Percent shrub cover was the only microsite vegetation characteristic that explained variation in diurnal temperature (simple linear regression; F_1,90_ = 6.50, P = 0.01, R^2^ = 0.06; Fig 5 inset). Thermal environments were cooler at successful nests than failed nests (S2 Fig; successful nests were 4°C cooler at 38°C), although, on average, temperatures of nests with different fates were similar; successful nests, on average, were cooler than failed nest: mean difference (±SE): 0.4±0.03. In total, three of the 14 failed nests had been depredated by mammals, while the cause of the other failed nests was not determined. Diurnal temperatures (09:00–19:00) above 46°C occurred at 6 of 9 successful (mean ± SE: 2.81 ± 0.64 hrs) and 11 of 14 failed (3.26 ± 0.35) nests. Thus, microclimates that correspond with egg mortality were common for both successful and failed nests. Successful and failed nest sites did not differ in standing dead vegetation height (ANOVA; F_1,21_ = 1.75, P = 0.2) or any other vegetation parameters (P > 0.05). Mean VOR (±SE) was 2.19 ± 0.38dm at successful nests (n = 9) and 1.89 ± 0.13dm at unsuccessful nests (n = 14).

Some variation among daytime T_iB_ in relation to landscape position was observed (S3 Fig). T_iB_ at landscape positions facing north approached average nest temperatures observed in this study. South-facing positions tended to be cooler than north-facing position; however, the difference was not significant (ANOVA; P = 0.2). Twelve of 23 nests were placed on non-southerly facing slopes (average slope declination: 19°), while the remainder were placed on south-facing slopes (declination: 15°). Nests were not placed on hilltops and only three of the 23 were located at the base of hills. Nest fate did not differ between nests placed in different landscape positions (P = 0.12).

### Within pasture nest habitat characteristics

The top model for predicting macrohabitat selection of nesting grouse included vegetation composition (AIC_*c*_ = 109.3, ω_i_ = 0.76) with the second-best model being the global model, ΔAIC_*c*_ = 3.53). Our preliminary analyses comparing model fit of linear vs. non-linear terms explaining habitat selection supported the inclusion of nonlinear terms (ΔAIC_*c* linear_ > 2.0; Table 2). The vegetation composition model was the only model in the 90% confidence set, which provided evidence for positive effect of percentage of shrub cover and standing dead vegetation at the nest and bare ground at moderate levels of 30–40% on nest microhabitat selection (Fig 5).

**Table 2:** Conditional logistic regression model rankings for predictors of Sharp-tailed grouse nest-site selection in the central Nebraska Sandhills, USA (2015–2016). Percent cover of bare ground, shrub, dead standing vegetation (dsv), litter depth (cm), maximum vegetation height (cm), and visual obstruction reading (VOR) were fixed effects included in candidate models. Nest ID was strata term.

Candidate Model	K^a^	AICc	ΔAICc	w_i_^b^
bare + bare ^2^ + shrub + shrub^2^ + dsv + dsv^2^	6	109.3	0.00	0.76
bare + bare ^2^ + shrub + shrub^2^ + dsv + dsv^2^ + litter + litter^2^ + VOR + VOR^2^ + height + height^2^	12	112.8	3.53	0.13
litter + litter^2^ + VOR +VOR^2^ + height + height^2^	6	114.3	5.04	0.06
litter + VOR + height	3	116.4	7.15	0.02
bare + shrub + dsv + litter + height	5	117.4	8.18	0.01
bare + shrub + dsv	3	117.7	8.44	0.01
null	0	149.0	39.78	0.00

^a^Number of parameters

^b^Akaike weight

### Future thermal habitat scenarios

**Fig 6:**
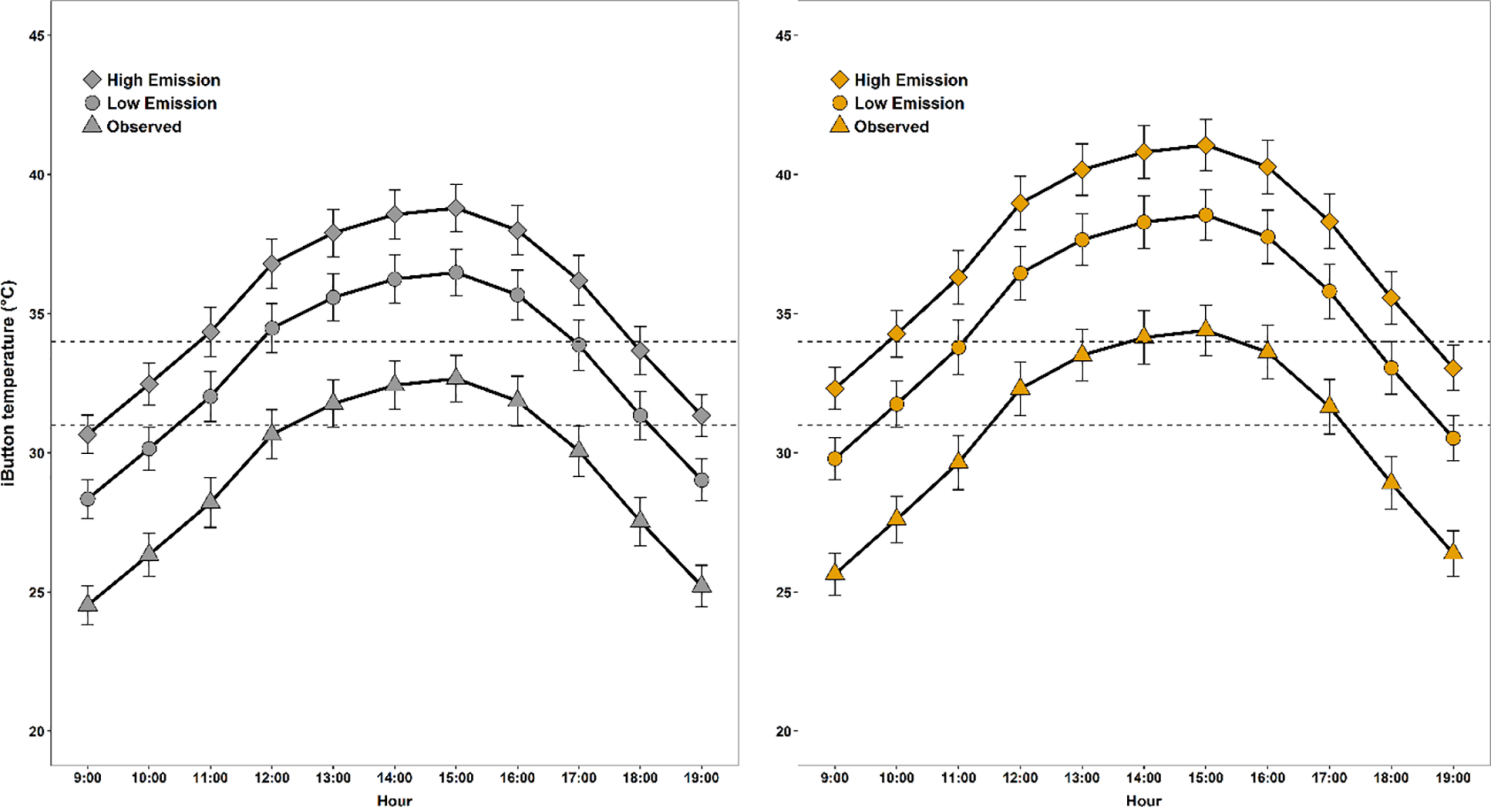
Predicted climate change imposes increased thermal constraint extent and strength on available nesting and landscape habitat. Modeled iButton temperature (T_iB_) (± SE) predicted at Sharp-tailed Grouse nest sites (A) and landscape sites (B) as a function of ambient temperature (T_air_). Marker shape indicates observed 2016 conditions (triangle) and T_air_ as projected by low (ring) and high (square) 2080 emission scenarios for Valentine, Nebraska, USA. The upper dotted line represents the temperature (34°C) at which prairie grouse daily nest survival probability decreases by 10% every half-hour and the lower dotted line represents the best-published estimate (31°C) at which gallinaceous birds are reported to exhibit thermal stress (i.e., gular fluttering).

T_air_ was closely related to temperatures measured at nest sites and landscape points (T_iB_, R^2^: 65% and 61%, respectively; Table 3). Models of T_iB_ associated with climate change suggest that nesting grouse will face greater T_iB_ for longer periods of the day (Fig 6). At the landscape level, we determined that future microclimate conditions during the nesting season could exceed T_iB_ of 31°C from 10:00–18:00 h for low and 09:00–19:00 h for high end of century emissions (Fig 6). While nest locations offered less extreme microclimatic conditions than the surrounding landscape, nests could be exposed to an increase in sub-optimal thermal conditions for longer periods of time. For example, during most of the daytime period except 13:00–16:00 h mean observed T_iB_ at nests remained lower than 31°C, which is a temperature associated with thermal stress. Nest temperatures will likely exceed 31°C during 7 daytime hours when exposed to potential low 2080 emission scenarios, while high emission scenarios may expose grouse nests to ≥ 31°C for at least 9 daytime hours (Fig 6). Nest sites chosen by female grouse in our study could be exposed to 34°C or greater for as long as 6 and 8 hours for low and high end of century emission scenarios, respectively.

**Table 3:** Model fit and parameter estimates (± SE) describing thermal environments at nest sites and landscape sites in the Nebraska Sandhills, Valentine, Nebraska, USA in 2016. Parameter abbreviations: T_air_ = air temperature.

Site Modeled	Intercept	T_air_	Fit (R^2^)
Nest*	-3.64 (± 0.56)	1.36 (± 0.02)	0.65
Landscape*	-4.95 (± 0.21)	1.48 (± 0.01)	0.61

* Indicates significance at a level of P < 0.01.

## Discussion

Our results demonstrate the interaction of a grouse with thermal heterogeneity inherent in a grassland landscape. The thermal environments of the Nebraska Sandhills provided a broad array of thermal conditions available to grouse. We found that T_iB_ differences reached up to 30°C among microclimates on the landscape during periods when ambient air temperature from our base weather station was greater than 35°C. We confirm the possibility for potential thermal extremes (≥ 60°C) on the grassland landscape, which underscores the significance of choices for Gallinaceous birds to acquire habitat offering favorable microclimates. Lower availability of microhabitats offering favorable thermal conditions would likely be a detriment to grouse fitness during times when air temperatures are non-conducive for survival. Rangeland management may maintain vegetation heterogeneity and thermally conducive environments, and our results suggest that nesting grouse benefit from thermally intricate matrices of small shrubs (e.g., soapweed yucca, *Yucca glauca*, wild rose, *Rosa sp*., and leadplant, *Amorpha canescens*) that may function as cover for nests (this study, [2, 44]).

We note that our interpretations assume T_iB_ in this study affects Sharp-tailed Grouse similarly as the species in the above studies [3, 21] and that T_iB_ reflects actual operative temperatures. Grouse nest sites provided less variable and cooler microclimate than conditions seen within the immediate vicinity. Furthermore, T_iB_ at nearby random locations exhibited substantial variation across the diel period and exhibited extreme heat potential; yet, nest locations mediated potential temperature extremes. The greatest contrast between nest site and landscape T_iB_ was observed on the warmest days (by an average of more than 4°C), indicating that sites offering thermal refuge likely helped in reducing heat stress for grouse during the nesting period. However, nest site selection may have been driven by concealment purposes and not solely a result of thermoregulatory needs and therefore a by-product of selection to reduce predation risk (e.g., [35]).

Studies in Oklahoma, U.S.A in the southern Great Plains, using a comparable approach, showed that bobwhite and Greater Prairie-Chicken exhibited similar interactions with thermal environs as grouse did in the Nebraska Sandhills. Specifically, nest sites moderated landscape microclimates, and nests that hatched exhibited cooler temperatures than failed nests [3, 21]. In our study, we determined hatched nest sites were exposed to 46°C, an egg-mortality inducing air temperature, for less time than failed nest sites. Such similarities indicate habitat selection by Gallinaceous birds in different grassland communities has a clear thermal component and emphasizes the significance of thermally-buffered sites for the persistence of Galliformes.

### Grassland management

We demonstrate that favorable microclimate space is limited now and in the future. Grouse should encounter increased exposure to unfavorable conditions during the breeding season with environmental climate change, which emphasizes the need for provision of favorable microclimates through planning for conservation of ground-dwelling species. The similar-sized Lesser Prairie-Chicken’s nest survival begins to drop by 10% for every half-hour above 34°C [25] and that 31°C is the air temperature documented for initiating evaporative cooling behaviors in Gallinaceous birds [46]. Additionally, our results provide new insight on the thermal ecology of Sharp-tailed Grouse nesting in low-intensity grazing lands. Such information may provide a baseline for understanding potential differences in the thermal ecology of this species in more heavily grazed systems. The lack of vegetation cover in intensively stocked grazing lands relative to grazing lands managed to maintain vegetation structure has recently been shown to depress survival in prairie grouse [68]. Yet, how variable stocking rates of grazing lands impacts prairie grouse thermal ecology remains to be tested. The grassland landscape at our study site was subject to high T_iB_ that exceeded 34°C during periods of the nesting season. Thus, our data demonstrate the value of shrubs and heterogeneity in structure of pastures, which is often not desired by managers for optimal beef production [69].

Habitat selection is known as an outcome from the interactive effects of abiotic conditions and vegetation structure that yield scale-dependent influences on organisms [3, 21, 29, 70]. Here we examined thermal conditions at locations selected by grouse for nesting and available habitat on the immediate landscape that aid in the understanding of the spatiotemporal heterogeneity of microclimates in this region. Nests in the Nebraska Sandhills were covered with vegetation cover to a greater extent than sites predominantly offered on the grassland landscape, and increasing shrub cover, for example, was associated with moderated thermal conditions. Further, we found a threshold for bare ground cover of 30% for within pasture habitat selection; suggesting dense vegetation cover is critical to grouse nesting habitat selection. In a similar study, Hovick, Elmore [21] found Greater Prairie-Chicken chose nest sites with greater vegetation cover around nests than sites less than 3 m from the selected nest site and successful nests were in cooler environments than failed nests. These results extend the notion that habitat selection in ground-nesting birds can be viewed through the lens of thermal environments and that microsites available in grassland landscapes can function to moderate thermal regimes [21]. Although grouse can withstand extreme temperatures and have evolved behavioral and physiological thermoregulatory mechanisms to cope with thermal extremes especially in colder environments [71, 72], our findings suggest that this species also select nest sites with a microclimate that likely reduce heat stress as well as nest predation. Increased temperatures have been linked to higher predation rates in two ways: increased activity of ectothermic nest predators (i.e., snakes; [73]) and decreased nest attentiveness by the parent which increases the opportunity for predators to detect nest locations [45].

### Future climate conditions

Global climate change may shift the placement of thermal regimes pertinent to organisms, especially at the resolution of the individual [16]; however, the spatial distribution and degree of magnitude of these likely shifts have received little attention (but see [74]). Temporal patterns in fine-scale thermal measurements (i.e., hourly or diurnal scales) can inform models for coarsely assessing bird populations’ response to climate [75–78], therefore, our findings are critical for such endeavors. Although our investigation did not use methods (i.e., nest cameras) to determine a direct connection between nest outcome and T_iB_ at the exact time that a nest failed [34], the stark T_iB_ maximal differences at nests of different fate suggest that further study is warranted. Given that temperatures >38°C can kill embryos if exposed for prolonged periods and ≥ 41°C compromises eggs at short time intervals [4], the importance of our findings for conservation efforts is elevated as regional temperatures are predicted to increase by at least 2.8°C over the next century [27]. Furthermore, adjustments in incubation behaviors can ameliorate impacts of hot microclimates on avian embryo and egg mortality [79], but this may occur at cost to the parent.

## Conclusions

Our major finding confirms that a favorable nest microclimate relative to nearby sites on the landscape and high vegetation cover are associated with grouse nest placement. We also confirmed thermal space for nesting grouse in the Nebraska Sandhills is likely to be reduced based on future climate scenarios. This finding was consistent with most previous research on the reproductive thermal ecology of Holarctic prairie grouse [21, 24]. In the Sand Shinnery Oak Prairies of New Mexico and Texas, Grisham, Godar [24] determined that Lesser Prairie-Chicken nests are more likely to experience failures due to nest microclimate conditions than populations further north in cooler and more humid regions of Kansas. Here we document the thermal properties of Nebraska Sandhills landscape features and grouse nests at the southern edge of their range in the central Great Plains. Grouse populations would benefit from research that identifies thermal landscapes at multiple scales across their range as well as land management techniques (e.g., prescribed fire, grazing, tree removal, herbicide application) that promote cooler, more humid microclimates for nesting and brood-rearing activities [21, 24, 38]. Such information at the local scale relevant to this species will enable us to determine how individuals operate in thermally heterogeneous landscapes [2, 19, 77, 78]. Although we show that grouse may respond to thermal heterogeneity in their environment by selecting habitats that moderate near ground climate for nesting, a broader effort to understand this behavior across their current range is needed. In addition, our work does not determine whether selection is driven by the need for nest concealment from predators or if microclimatic differences between nests and nearby available sites is simply a by-product of predation-limiting choices. Further work on this question is needed. The incorporation of comprehensive assessments of fine-scale thermal conditions as components of the experimental framework would allow investigations to associate regional macroclimate to the actual microclimates that individuals experience.

## Supporting information

S1 Fig Thermal environments on the grassland landscape are decoupled from nest sites through an increase in intensification of heat. Differences (TiB—T_air_) between diurnal iButton temperature (T_iB_) and ambient air temperature (T_air_) (± SE) recorded from the daytime period (09:00–19:00 h) at Sharp-tailed Grouse nests (n = 23) and landscape points (n = 116) at Valentine, Nebraska, USA in May to July 2016.
(DOCX)

S2 Fig Successful nest sites moderated thermal conditions more than unsuccessful nests during high heat. Modeled iButton temperatures at successful and failed nests of Sharp-tailed Grouse in the Nebraska Sandhills, Valentine, Nebraska in June 2016 recorded during the full sampling period (0:00–24:00 h). Successful nests experienced temperatures that were 4°C cooler at 38°C.
(DOCX)

S3 Fig Sandhill topography slightly varied in thermal environments. Boxplots of mean iButton temperatures recorded from 0900–1900 along different topographic positions on the landscape grid from May to July 2016 sites near Valentine, Nebraska, USA. Bottom dashed line is mean nest T_iB_ and top dashed line is mean T_iB_ at nearby random microsites.
(DOCX)

S1 Data Temperature, vegetation, and nest success data for 23 nests, 69 random microsites, and 60 landscape sites used to determine 2016 thermal environments and nest vegetation characteristics available to Sharp-tailed grouse in the Nebraska Sandhills, U.S.A. Pasture-scale vegetation data for nest site selection analysis for 2015 and 2016 are included.
(XLSX)
