# Supplementary material for: Present and future thermal environments available to Sharp-tailed Grouse in an intact grassland
Source: PLoS One. 2018 Feb 7;13(2):e0191233. doi: 10.1371/journal.pone.0191233 (PMC5802491; doi:10.1371/journal.pone.0191233)
Supplement: S1 Fig — Differences (TiB—Tair) between diurnal iButton temperature (TiB) and ambient air temperature (Tair) (± SE) recorded from the daytime period (09:00–19:00 h) at Sharp-tailed Grouse nests (n = 23) and landscape points (n = 116) at Valentine, Nebraska, USA in May to July 2016. (DOCX) [file pone.0191233.s001.docx]

**
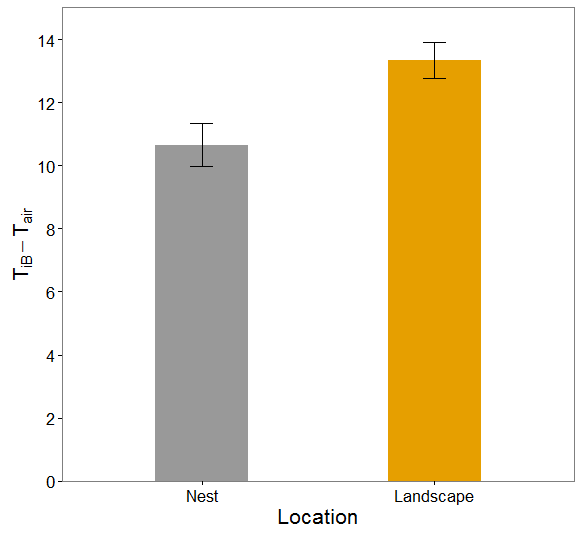
**

**S1 Fig. Thermal environments on the grassland landscape are decoupled from nest sites through an increase in intensification of heat.** Differences (T_iB_ - T_air_) between diurnal iButton temperature (T_iB_) and ambient air temperature (T_air_) (± SE) recorded from the daytime period (09:00-19:00 h) at Sharp-tailed Grouse nests (n = 23) and landscape points (n = 116) at Valentine, Nebraska, USA in May to July 2016.
