# Supplementary material for: Present and future thermal environments available to Sharp-tailed Grouse in an intact grassland
Source: PLoS One. 2018 Feb 7;13(2):e0191233. doi: 10.1371/journal.pone.0191233 (PMC5802491; doi:10.1371/journal.pone.0191233)
Supplement: S2 Fig — Modeled iButton temperatures at successful and failed nests of Sharp-tailed Grouse in the Nebraska Sandhills, Valentine, Nebraska in June 2016 recorded during the full sampling period (0:00–24:00 h). Successful nests experienced temperatures that were 4°C cooler at 38°C. (DOCX) [file pone.0191233.s002.docx]

**
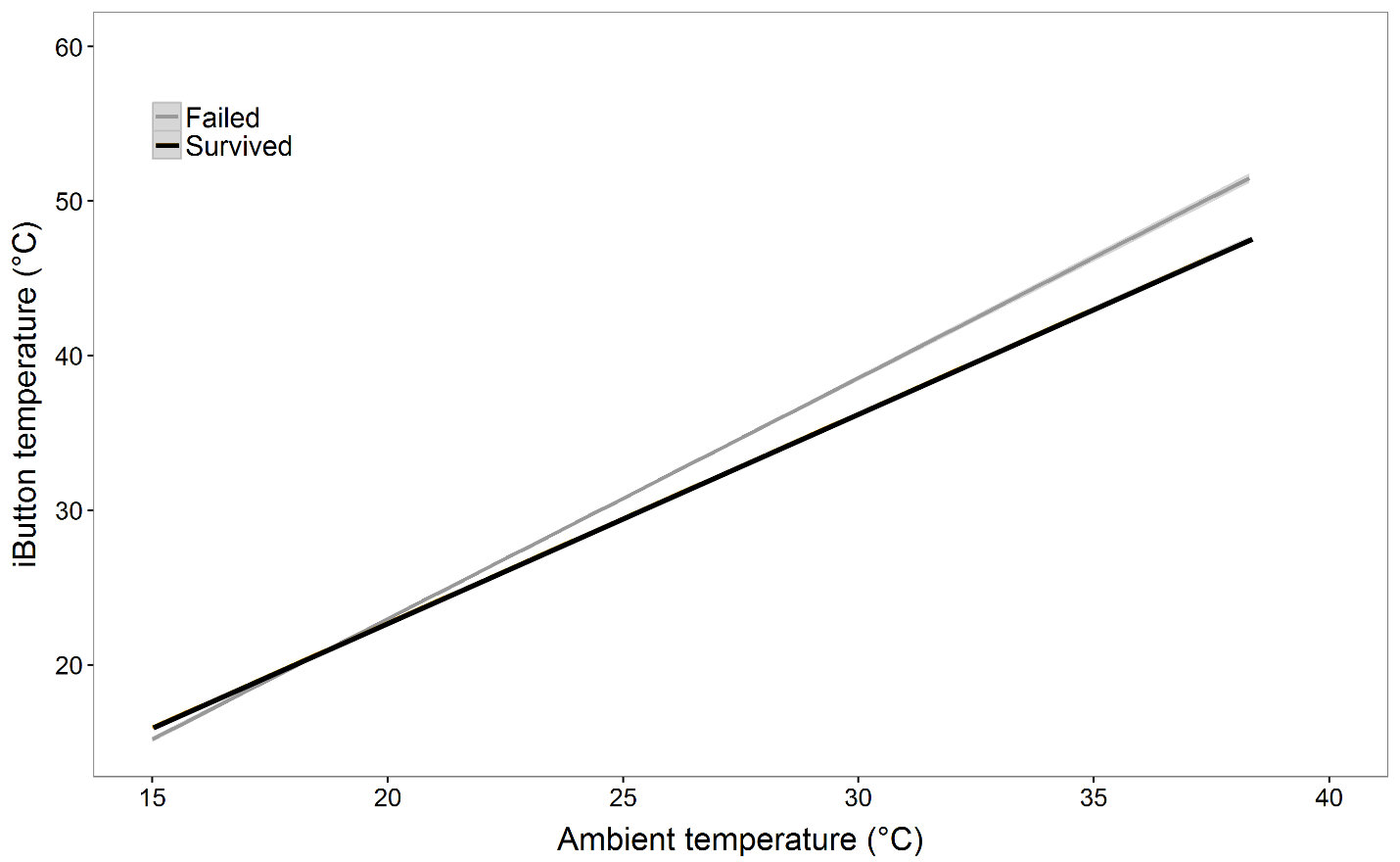
**

**S2 Fig. Successful nest sites moderated thermal conditions more than unsuccessful nests during high heat.** Modeled iButton temperatures at successful and failed nests of Sharp-tailed Grouse in the Nebraska Sandhills, Valentine, Nebraska in June 2016 recorded during the full sampling period (0:00-24:00 h). Successful nests experienced temperatures that were 4°C cooler at 38°C.
