# Supplementary material for: Present and future thermal environments available to Sharp-tailed Grouse in an intact grassland
Source: PLoS One. 2018 Feb 7;13(2):e0191233. doi: 10.1371/journal.pone.0191233 (PMC5802491; doi:10.1371/journal.pone.0191233)
Supplement: S3 Fig — Boxplots of mean iButton temperatures recorded from 0900–1900 along different topographic positions on the landscape grid from May to July 2016 sites near Valentine, Nebraska, USA. Bottom dashed line is mean nest TiB and top dashed line is mean TiB at nearby random microsites. (DOCX) [file pone.0191233.s003.docx]

**
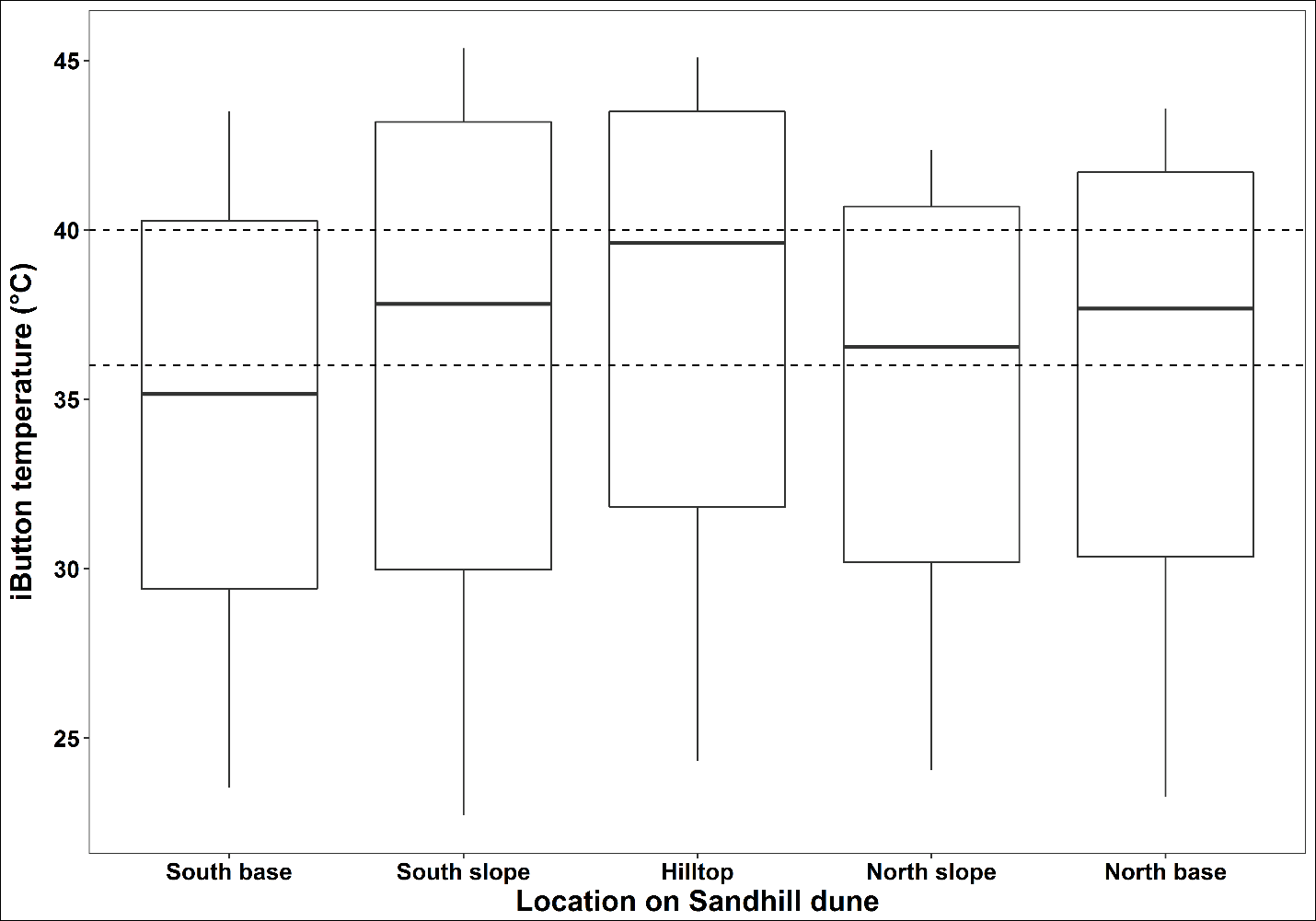
**

**S3 Fig. Sandhill topography slightly varied in thermal environments.** Boxplots of mean iButton temperatures recorded from 0900-1900 along different topographic positions on the landscape grid from May to July 2016 sites near Valentine, Nebraska, USA. Bottom dashed line is mean nest T_iB_ and top dashed line is mean T_iB_ at nearby random microsites.
